# Supplementary material for: Read trimming has minimal effect on bacterial SNP-calling accuracy
Source: Microb Genom. 2020 Dec 17;6(12):mgen000434. doi: 10.1099/mgen.0.000434 (PMC8116680; doi:10.1099/mgen.0.000434)
Supplement: Supplementary material 1 [file mgen-6-434-s001.pdf]

Supplementary Figures

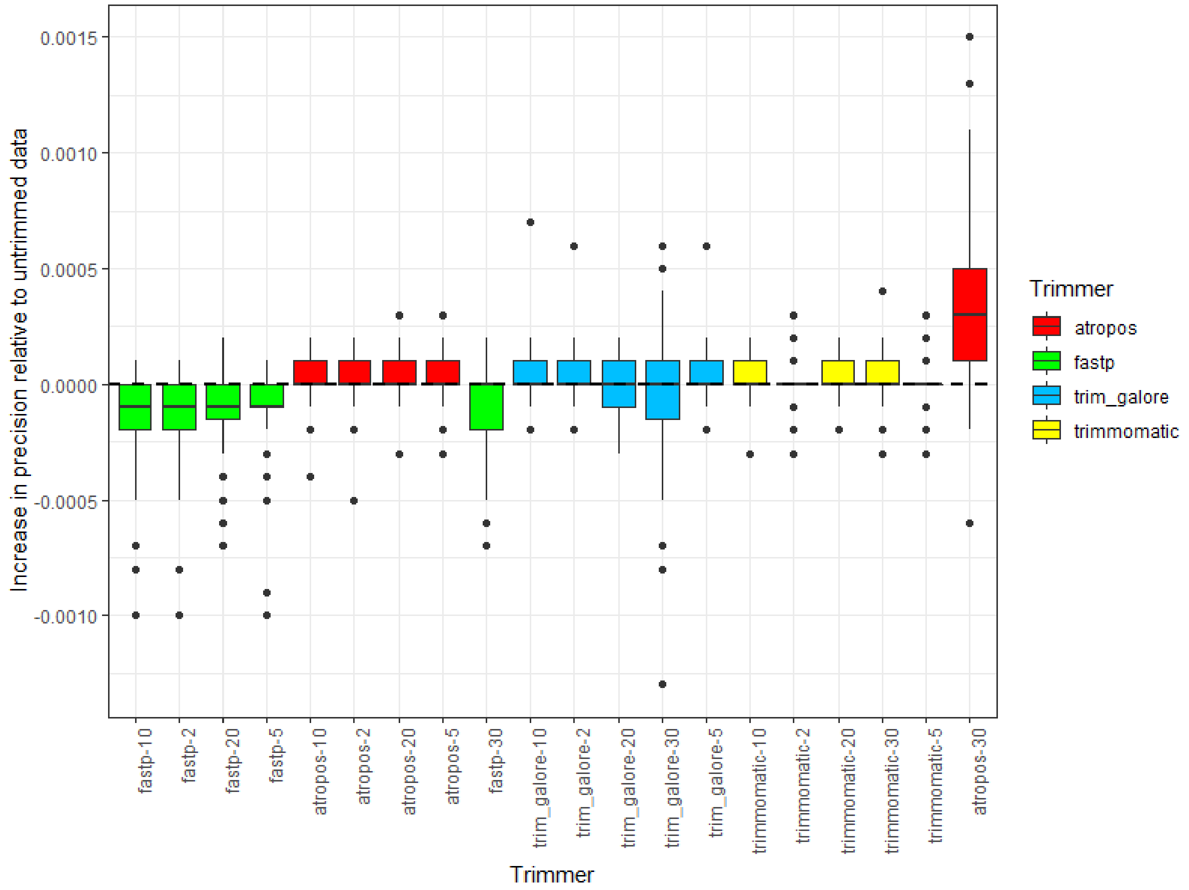

Supplementary Figure 1. Effect of read trimming upon precision (positive predictive value) when SNP calling in a curated Gram-negative dataset. Median difference in precision per trimmer relative to untrimmed data, across a range of trimming stringencies (i.e. varying the Phred score threshold for trimming 3' bases). Boxes represent the interquartile range of precision, with midlines representing the median. Upper and lower whiskers extend, respectively, to the largest and smallest values no further than 1.5x the interquartile range. Data beyond the ends of each whisker are outliers and plotted individually. Columns are ordered according to median precision and coloured according to the trimmer used. The dashed line y=0 is marked in black. The raw data for this figure is available in **Supplementary Table 5**. Note that fastp implements quality filters other than 3' trimming by default, which for the data in this figure were retained. A version of this figure with these filters disabled is available in **Supplementary Figure 3**.

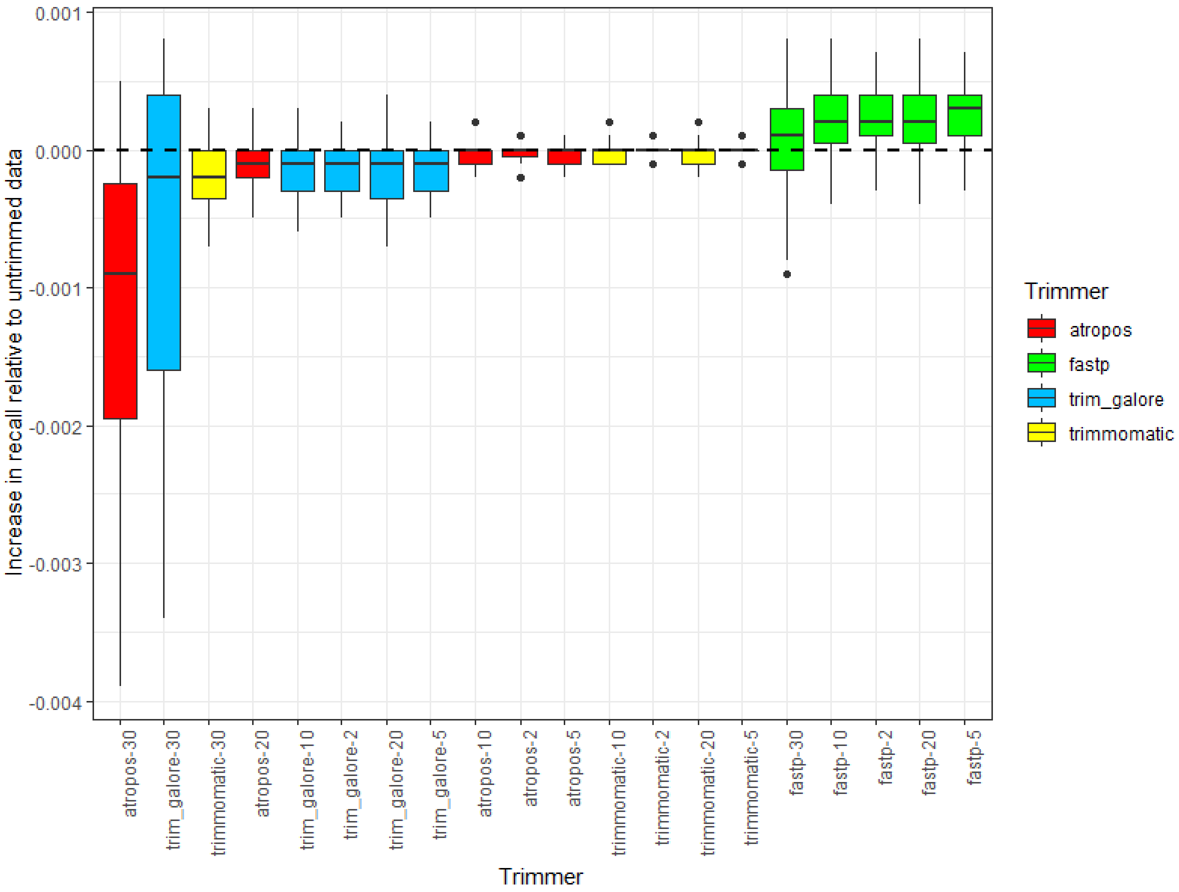

Supplementary Figure 2. Effect of read trimming upon recall (sensitivity) when SNP calling in a curated Gram-negative dataset.

Median difference in recall per trimmer relative to untrimmed data, across a range of trimming stringencies (i.e. varying the Phred score threshold for trimming 3’ bases). Boxes represent the interquartile range of recall, with midlines representing the median. Upper and lower whiskers extend, respectively, to the largest and smallest values no further than 1.5x the interquartile range. Data beyond the ends of each whisker are outliers and plotted individually. Columns are ordered according to median recall and coloured according to the trimmer used. The dashed line y=0 is marked in black. The raw data for this figure is available in **Supplementary Table 5**. Note that fastp implements quality filters other than 3’ trimming by default, which for the data in this figure were retained. A version of this figure with these filters disabled is available in **Supplementary Figure 3**.

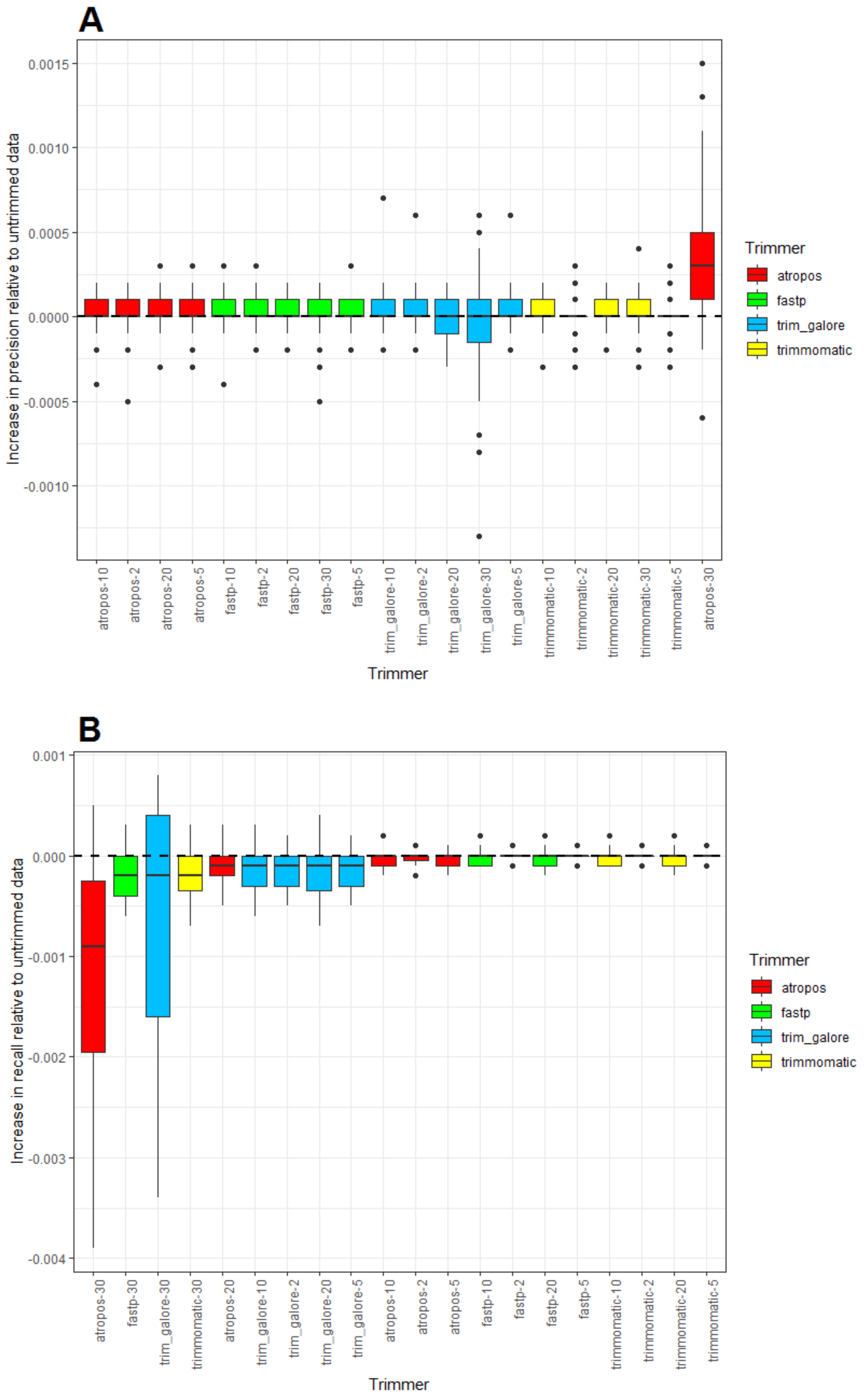
A
Increase in precision relative to untrimmed data
0.0015
0.0010
0.0005
0.0000
-0.0005
-0.0010
atropos-10
atropos-2
atropos-20
atropos-5
fastp-10
fastp-2
fastp-20
fastp-30
fastp-5
trim_galore-10
trim_galore-2
trim_galore-20
trim_galore-30
trim_galore-5
trimmomatic-10
trimmomatic-2
trimmomatic-20
trimmomatic-30
trimmomatic-5
atropos-30
Trimmer
Trimmer
atropos
fastp
trim_galore
trimmomatic
B
Increase in recall relative to untrimmed data
0.001
0.000
-0.001
-0.002
-0.003
-0.004
atropos-30
fastp-30
trim_galore-30
trimmomatic-30
atropos-20
trim_galore-10
trim_galore-2
trim_galore-20
trim_galore-5
atropos-10
atropos-2
atropos-5
fastp-10
fastp-2
fastp-20
fastp-5
trimmomatic-10
trimmomatic-2
trimmomatic-20
trimmomatic-5
Trimmer
Trimmer
atropos
fastp
trim_galore
trimmomatic

31

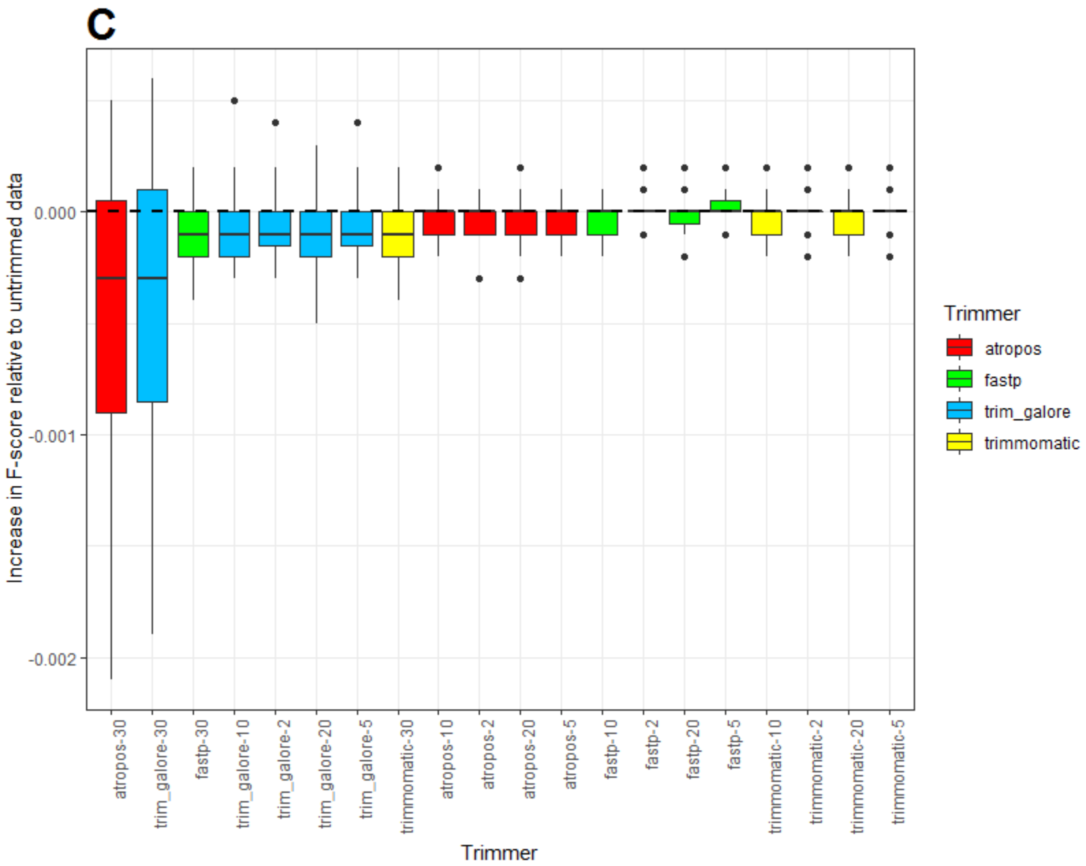

Supplementary Figure 3. Effect of read trimming upon precision (positive predictive value), recall (sensitivity) and F-score when SNP calling in a curated Gram-negative dataset, disabling fastp default options.

The data shown in this figure is as shown in **Figure 1** (F-score), **Supplementary Figure 1** (precision) and **Supplementary Figure 2** (recall), with the exception of values for fastp, in which the default trimming option ('qualified quantity', requiring that > 40% of the bases in each read have Phred > 15) is now explicitly disabled and the trimming performed is limited only to adapter removal and 3' quality trimming.

This figure shows that across all four trimmers, 3' quality-trimming, in general, marginally increases precision (panel A) but at the expense of recall (panel B) and thereby overall F-score (panel C). Panels show the median difference in precision (A), recall (B) or F-score (C) per trimmer relative to untrimmed data, across a range of trimming stringencies (i.e. varying the Phred score threshold for trimming 3' bases). Boxes represent the interquartile range of precision (A), recall (B) and F-score (C), with midlines representing the median. Upper and lower whiskers extend, respectively, to the largest and smallest values no further than 1.5x the interquartile range. Data beyond the ends of each whisker are outliers and plotted individually. Columns are ordered according to median precision (A), recall (B) and F-score

(C), and coloured according to the trimmer used. The dashed line y=0 is marked in black. The raw data for this figure is available in **Supplementary Table 5**.

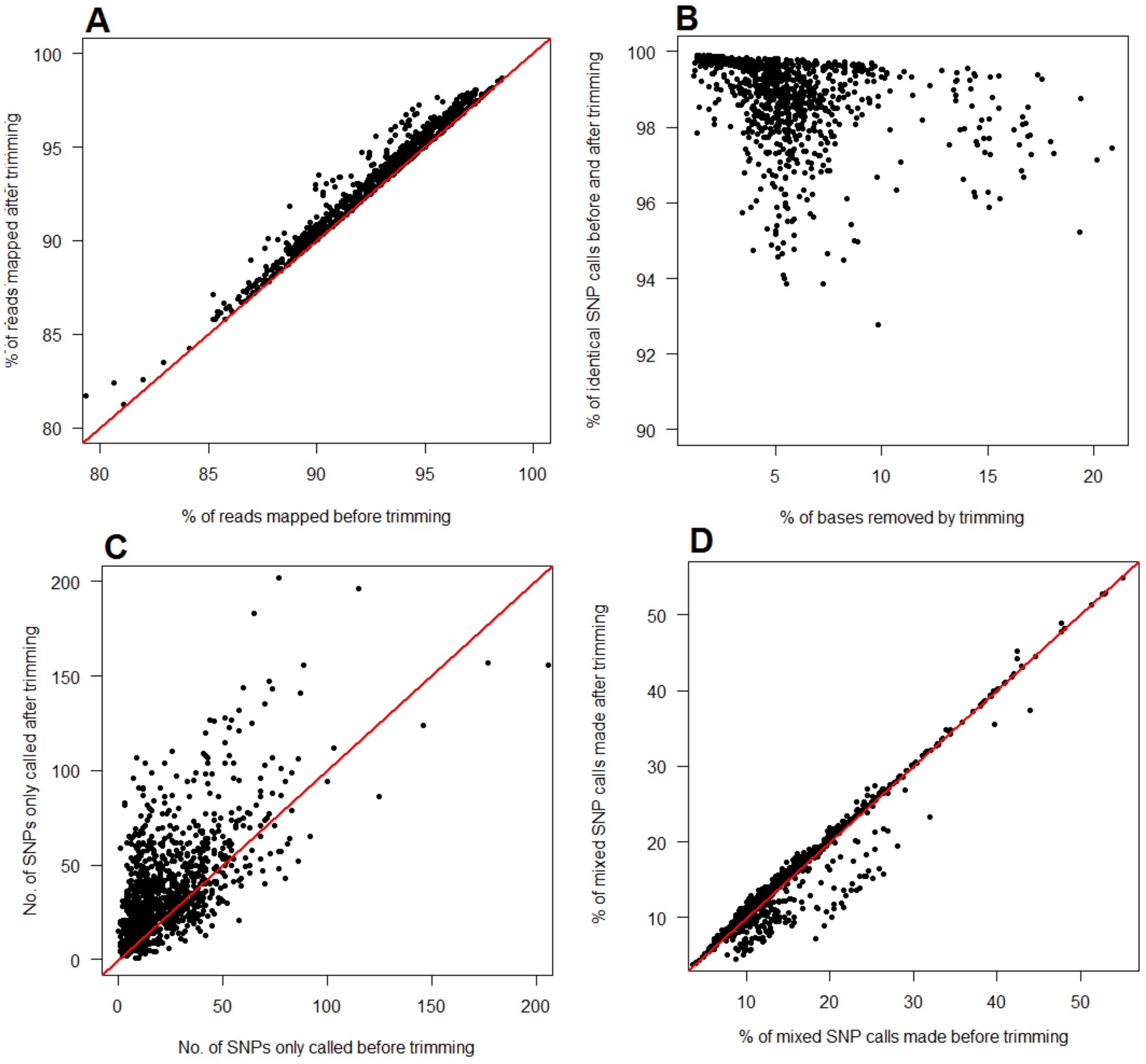

Supplementary Figure 4. Effect of read trimming upon SNP calls made using publicly-archived *S. aureus* sequencing data.

This figure recapitulates patterns seen in **Figure 2** and illustrates the effect of read trimming upon SNP calls made in *S. aureus*. Trimming marginally increases the proportion of successfully aligned reads, albeit from a high baseline value, > 85% (panel A). The majority of SNPs (> 96%) are nevertheless called irrespective of any trimming (panel B). A relatively small number of SNPs (often < 200) are only called when using either raw, or trimmed data, but not both (panel C). The proportion of mixed SNP calls, considered a proxy of false positive calling, decreases when using trimmed data (panel D). The raw data for this figure is available in **Supplementary Table 8** and represents 1100 *S. aureus* samples, with a mean of 15,167 SNPs per sample. The red line denotes y = x.

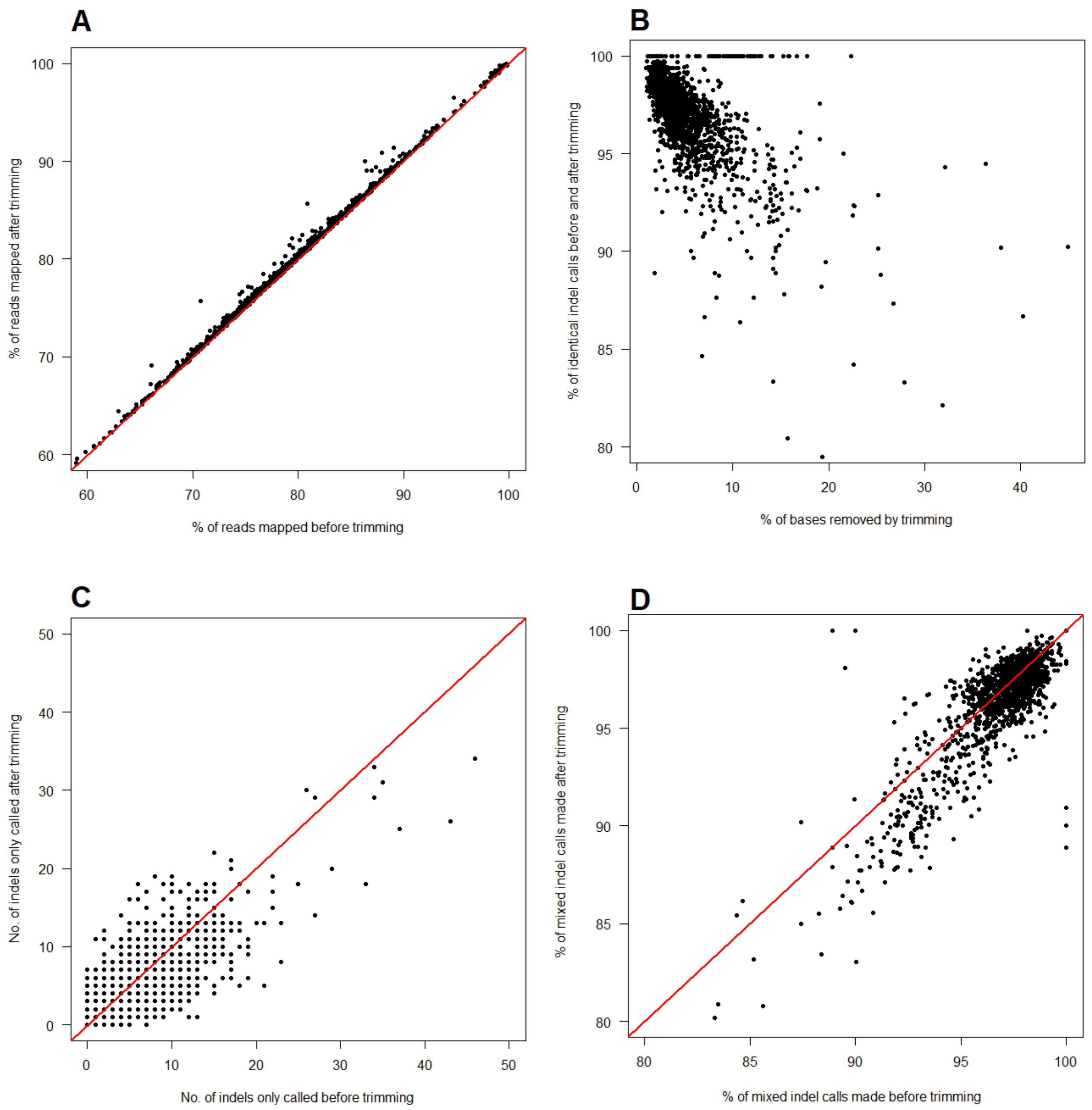

Supplementary Figure 5. Effect of read trimming upon indel calls made using publicly-archived *E. coli* sequencing data.

Trimming marginally increases the proportion of successfully aligned reads (panel A) although the interpretation of those alignments (i.e. indel calling) is not substantially altered, with the majority of indel (> 95%) called irrespective of trimming (panel B). A relatively small number of indels (in the majority of cases, < 20) are only called when using either raw, or trimmed data, but not both (panel C). The proportion of mixed indel calls, considered a proxy of false positive calling, decreases when using trimmed data although is in general rather high (panel D). The raw data for this figure is available in **Supplementary Table 6**

and represents 1606 *E. coli* samples, with a mean of 316 indels per sample. The red line denotes y = x.

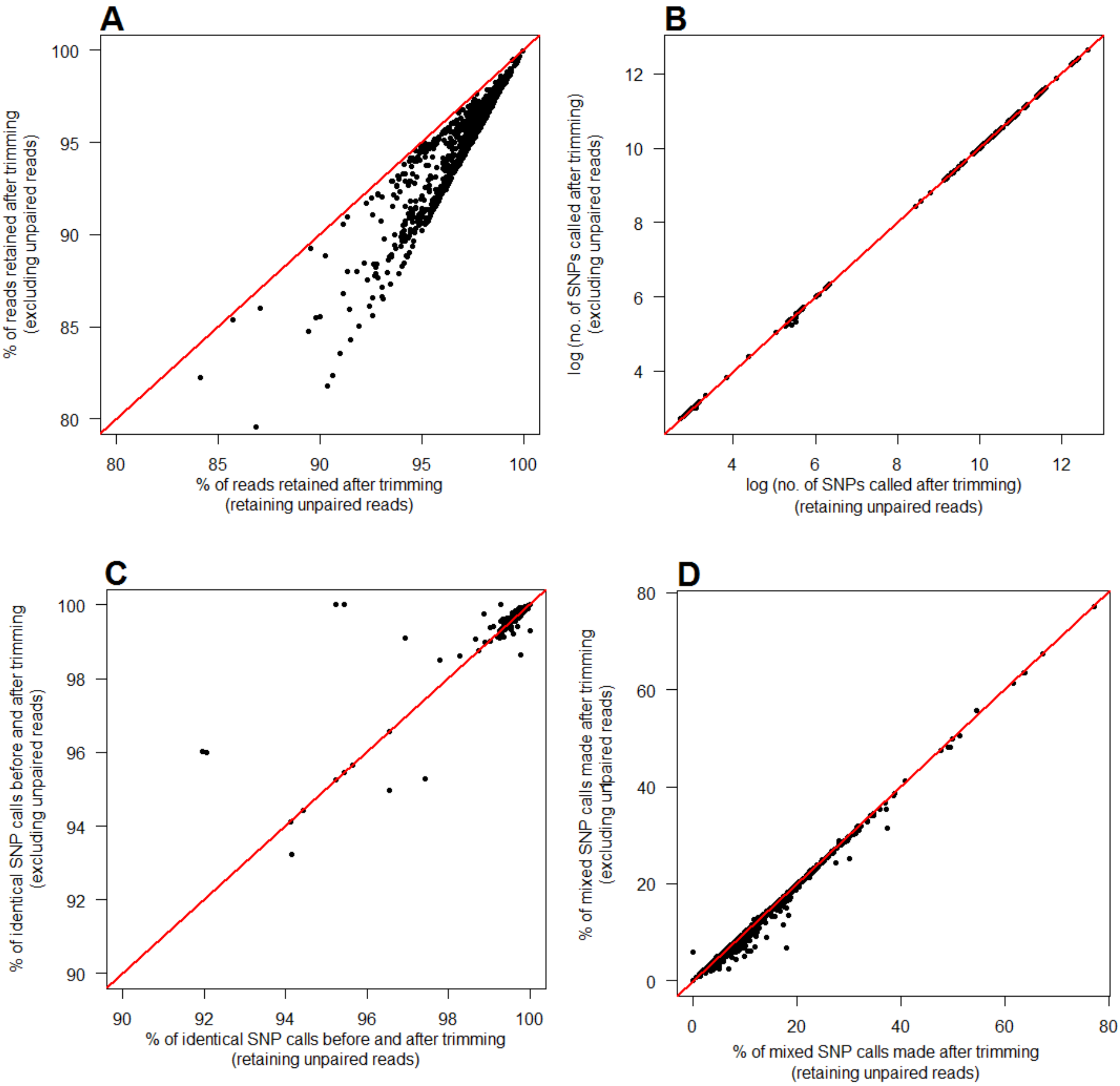

Supplementary Figure 6. Discarding unpaired reads after trimming has negligible impact on the number of SNPs called in *E. coli*.

This figure illustrates the effect of read trimming upon SNP calls made in *E. coli*, with the read trimmer retaining or discarding unpaired reads (unpaired reads are those when one end of a pair is discarded by the trimmer, and so are output as *de facto* single-end; SE reads). While discarding SE reads reduces, by definition, the proportion of reads available for mapping and SNP calling, this only represents a substantial proportion (> 5%) of the total in limited cases (panel A). There is little discernible difference both in the absolute number of SNPs called with and without SE reads (panel B) and the percentage of SNPs identically called before and after trimming (panel C), although seemingly a small decrease in the number of mixed calls (a proxy of false positives) made when discarding the unpaired reads

(panel D). Raw data for this figure is available in **Supplementary Table 6** and represents 1606 *E. coli* samples. The red line denotes y = x.

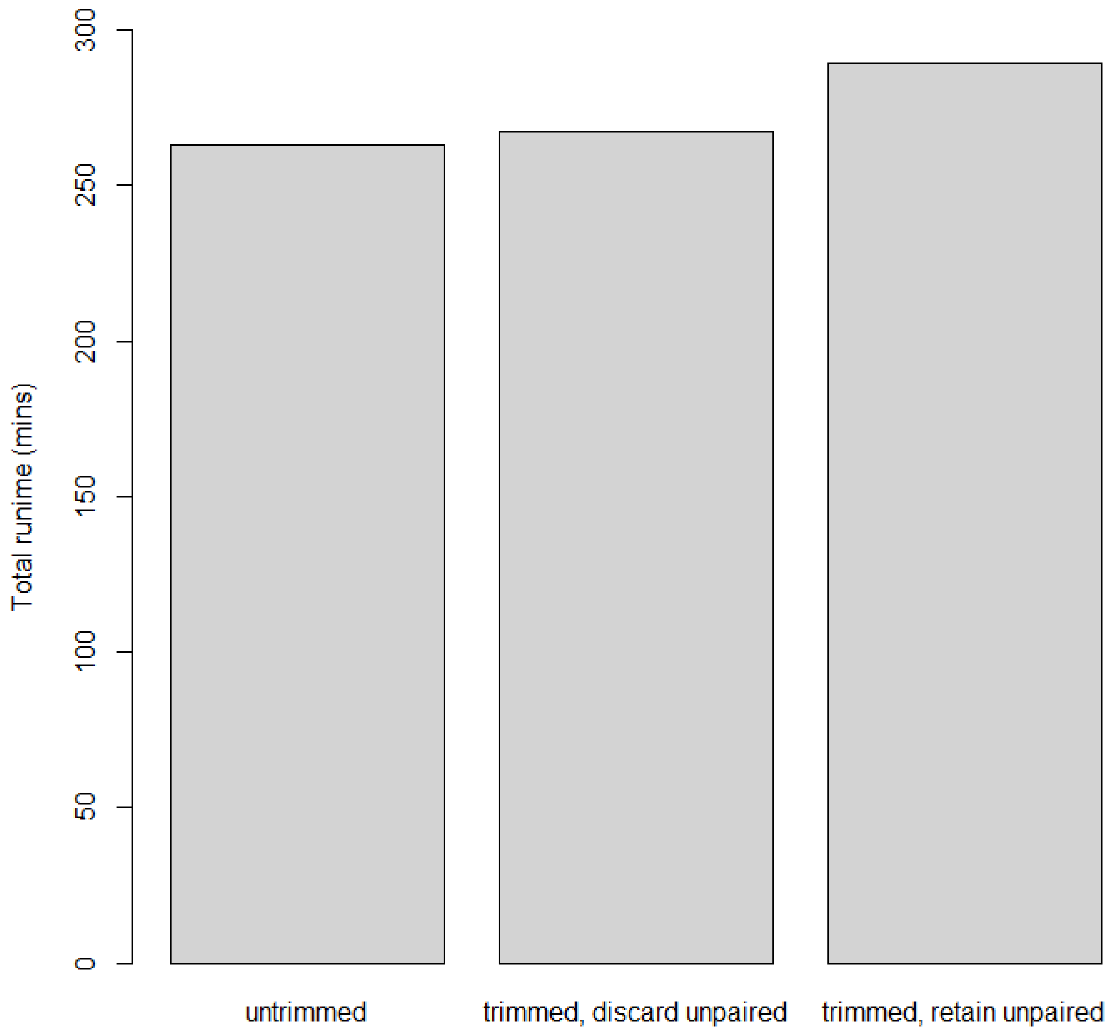

Supplementary Figure 7. Total runtime of SNP calling pipelines that either trim or do not trim the input reads.

This figure shows the total clock time taken by three BWA-mem/mpileup SNP calling pipelines when processing the Gram-negative dataset, either (a) omitting the read trimming step, (b) running fastp with trailing Q < 20 and retaining reads unpaired by trimming, and (c) running fastp with trailing Q < 20 and discarding reads unpaired by trimming.

For the purpose of this analysis, the 17 sets of reads comprising this dataset were processed serially. Furthermore, while the core constituents of the SNP calling pipeline (fastp, BWA, mpileup) can be multithreaded, each program was executed using 1 thread on a server comprising 20 2.2GHz Intel Xeon CPUs and running Ubuntu v5.4.0-6. The figure demonstrates that while trimming with fastp is rapid (as detailed in **Supplementary Table 5**,

each of the 17 samples comprises 1-2 million reads, which are processed in seconds), the division of trimmed reads into ‘paired’ and ‘unpaired’ subsets necessitates running the alignment step twice. In this figure, the retention of unpaired reads adds 27 minutes additional runtime, approximately 10% of the total runtime of the ‘untrimmed’ pipeline.

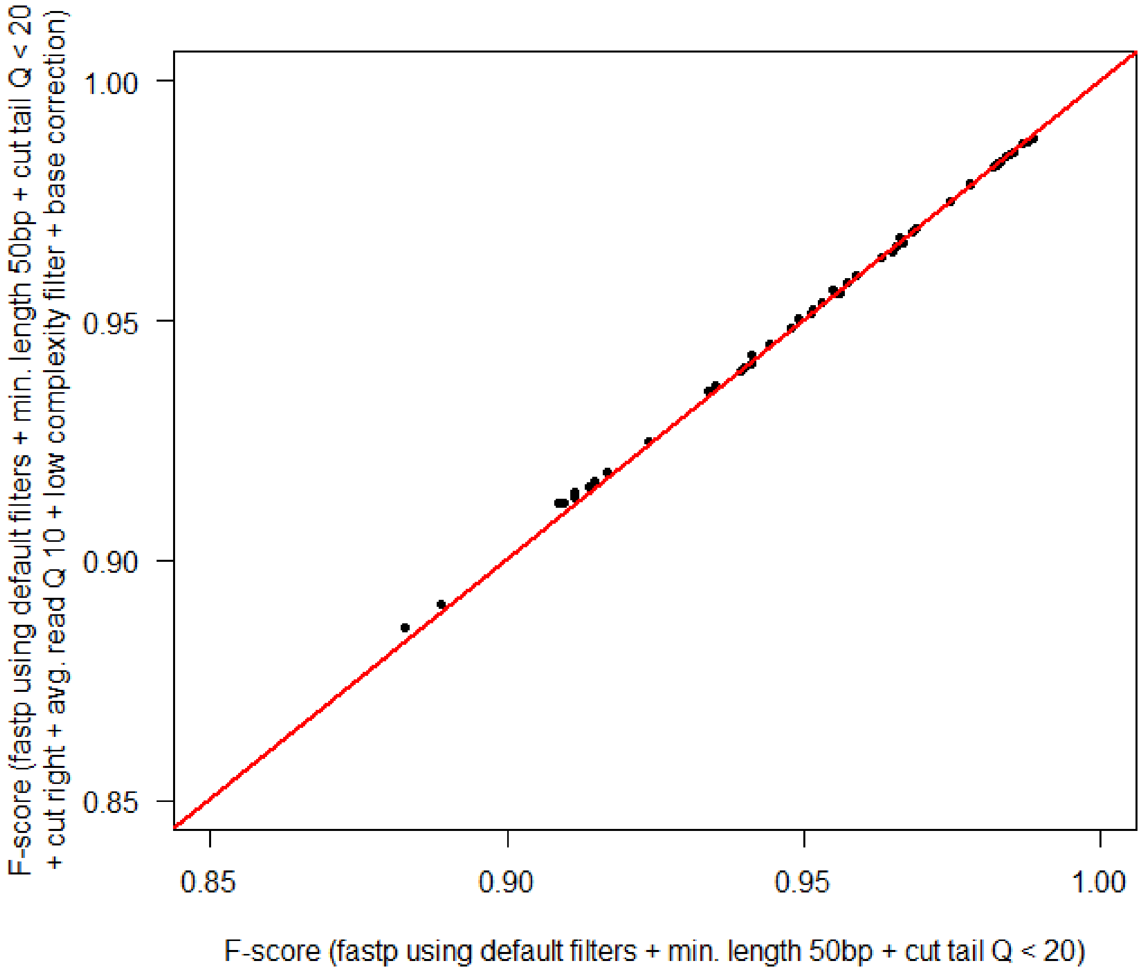

Supplementary Figure 8. Pre-processing with additional filters has negligible effect on F-score when SNP calling in a curated Gram-negative dataset.

This figure shows the F-score per aligner/caller combination when SNP calling each of 17 Gram-negative genomes, after pre-processing reads using fastp with two sets of parameters: one simple, one complex. The x-axis applies a simple set of filters: the two default settings of adapter-trimming and a minimum 'qualified quantity' of bases, plus 3' quality trimming ('cut tail') and a minimum read length of 50bp. The y-axis supplements these filters with four more: 'cut left' (cutting reads should the mean quality within a 4bp window, advanced 5' to 3', fall below 20), 'low complexity' (requiring that 30% or more of the bases in each read are followed by a different base), 'average quality' (requiring a mean base quality across the entire read of > 10), and 'correction' (correcting mismatched base pairs in regions of paired end reads that overlap each other, if one base has a quality higher than the other [by default, requiring a minimum overlap of 30bp, a difference in base qualities of 5, and no more than 20% of the bases in the overlapping region needing correction]). The raw data for this figure

is available in **Supplementary Table 5**. Note that the pipeline used for SNP calling applied VCF filter criteria to reduce errors. An analysis of the effect the additional fastp parameters have upon SNP calling, irrespective of VCF filtering, is given in **Supplementary Figure 9**.

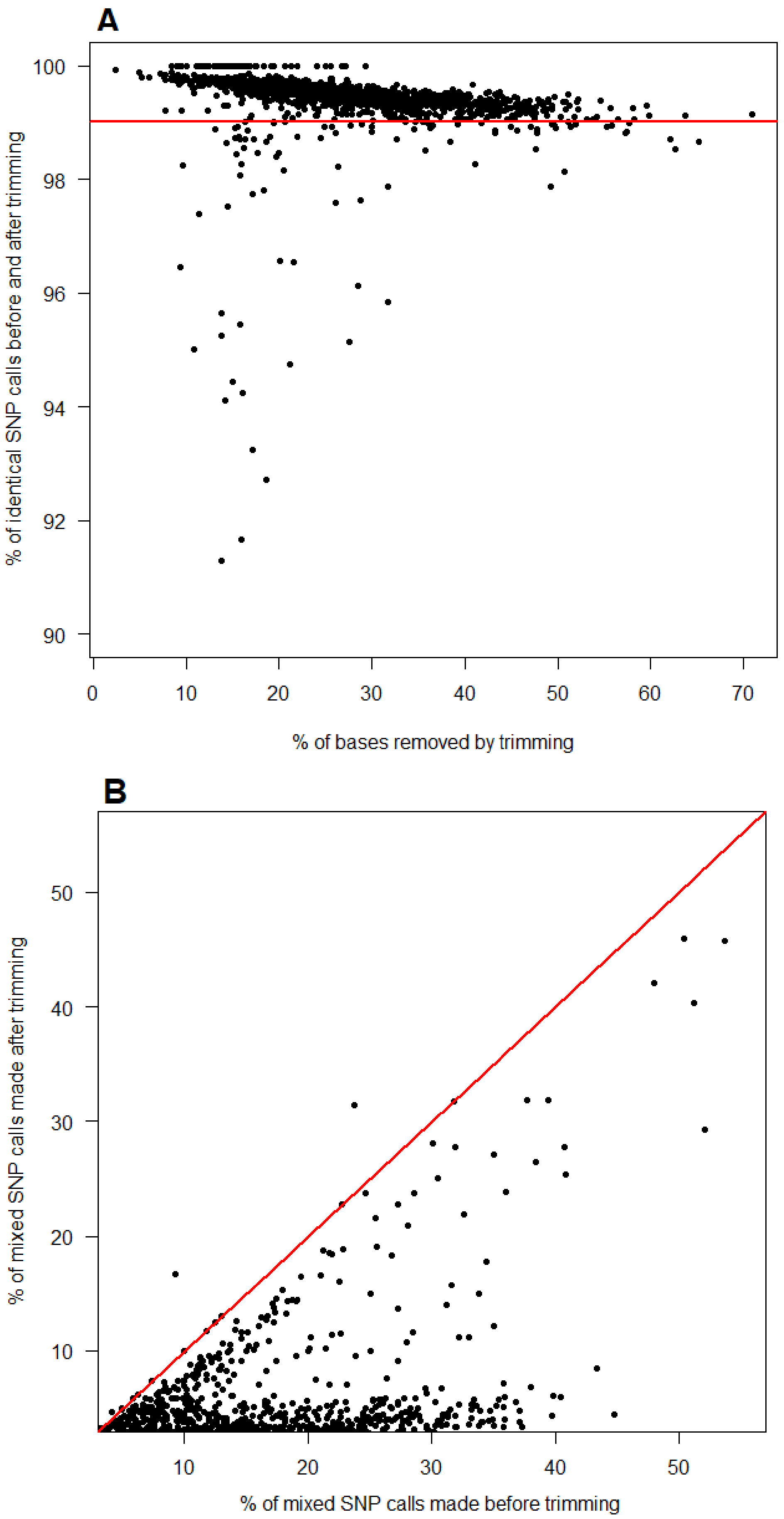
A
% of identical SNP calls before and after trimming
100
98
96
94
92
90
0
10
20
30
40
50
60
70
% of bases removed by trimming
B
% of mixed SNP calls made after trimming
50
40
30
20
10
10
20
30
40
50
% of mixed SNP calls made before trimming

Supplementary Figure 9. Pre-processing with additional filters makes little difference to the set of SNPs called in *E. coli*, but does significantly reduce the number of mixed calls. This figure shows the effect of trimming upon SNP calling when using fastp with multiple parameters: adapter-trimming, a minimum 'qualified quantity' of bases, 3' quality trimming ('cut tail'), sliding window trimming ('cut left'), a minimum read length of 50bp, a minimum read complexity, and base correction (parameters detailed more fully in **Supplementary Figure 8**). The vast majority of SNPs (> 99% in 1502 of the 1606 samples) are identically called irrespective of trimming (panel A). However, the proportion of mixed calls, considered a proxy of false positives, decreases significantly when using trimmed data (panel B; the median percentage of mixed SNP calls made before and after trimming are 10.24% and 2.63%, respectively; Mann Whitney U $p < 2.2 \times 10^{-16}$). The raw data for this figure is available in **Supplementary Table 6** and represents 1606 *E. coli* samples, with a mean of 64,536 SNPs per sample. The red lines denotes y = 99% (panel A) and y = x (panel B).

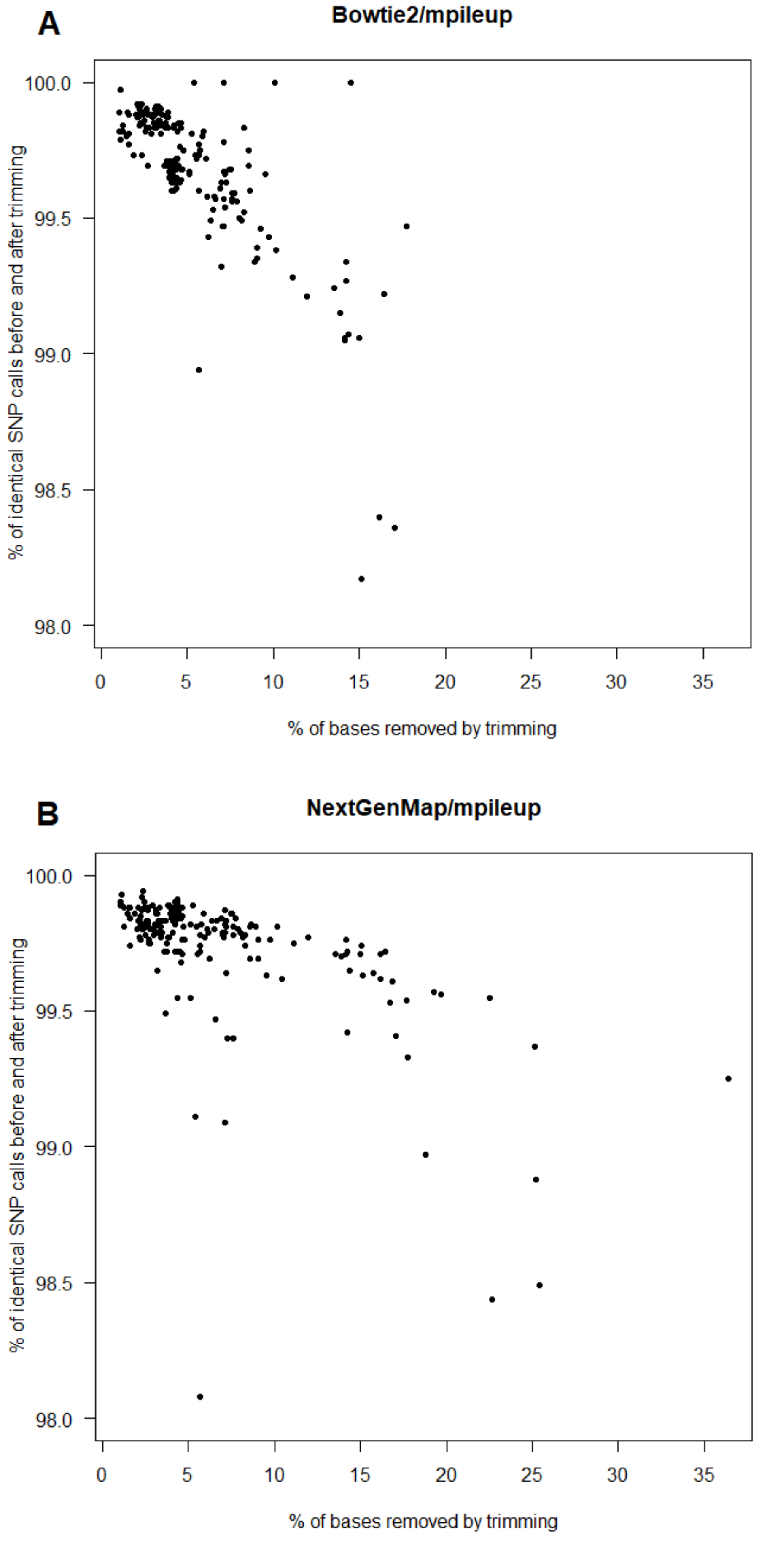
A
Bowtie2/mpileup
% of identical SNP calls before and after trimming
100.0
99.5
99.0
98.5
98.0
0
5
10
15
20
25
30
35
% of bases removed by trimming
B
NextGenMap/mpileup
% of identical SNP calls before and after trimming
100.0
99.5
99.0
98.5
98.0
0
5
10
15
20
25
30
35
% of bases removed by trimming

Supplementary Figure 10. Effect of read trimming upon SNP calls made using publicly-archived *E. coli* sequencing data and two different SNP-calling pipelines.

This figure shows that irrespective of SNP calling pipeline, the majority of SNPs are identically called irrespective of whether trimmed or untrimmed reads are used as input. Panels A and B show results from two different pipelines (Bowtie2/mpileup and NextGenMap/mpileup, respectively) and can be contrasted with **Figure 2B**, which shows similar results obtained using the principal pipeline employed in this study, BWA-mem/mpileup. The data represents a subset of the first 200 *E. coli* samples from a full set of 1606, in alphabetical order of SRA sample ID. Raw data for this figure is available in **Supplementary Table 6**. > 99% of SNPs are identically called, irrespective of trimming, in 179 and 190 of 200 samples when using Bowtie2/mpileup and NextGenMap/mpileup, respectively. With Bowtie2, there is a sharper decline in the percentage of identically called SNPs when a higher number of bases have been trimmed. This may be attributed to the nature of the alignment algorithm. Unlike NextGenMap, which 'soft clips' reads, Bowtie2 attempts to align every base of a read. As such, it should be more greatly affected by read trimming which, by removing lower-quality bases, ensures a read is more likely to successfully align.
